# Generation of Microplastics from Biodegradable Packaging Films Based on PLA, PBS and Their Blend in Freshwater and Seawater

**DOI:** 10.3390/polym16162268

**Published:** 2024-08-10

**Authors:** Annalisa Apicella, Konstantin V. Malafeev, Paola Scarfato, Loredana Incarnato

**Affiliations:** Department of Industrial Engineering, University of Salerno, Via Giovanni Paolo II n. 132, 84084 Fisciano, SA, Italy; anapicella@unisa.it (A.A.); kmalafeev@unisa.it (K.V.M.); lincarnato@unisa.it (L.I.)

**Keywords:** biodegradable microplastics, PBS/PLA blend, degradation, fresh water, seawater

## Abstract

Biodegradable polymers and their blends have been advised as an eco-sustainable solution; however, the generation of microplastics (MPs) from their degradation in aquatic environments is still not fully grasped. In this study, we investigated the formation of bio-microplastics (BMPs) and the changes in the physicochemical properties of blown packaging films based on polylactic acid (PLA), polybutylene succinate (PBS) and a PBS/PLA 70/30 wt% blend after degradation in different aquatic media. The tests were carried out in two temperature/light conditions to simulate degradation in either warm water, under sunlight exposure (named Warm and Light—W&L), and cold deep water (named Cold and Dark—C&D). The pH changes in the aqueous environments were evaluated, while the formed BMPs were analyzed for their size and shape alongside with variations in polymer crystallinity, surface and mechanical properties. In W&L conditions, for all the films, the hydrolytic degradation led to the reorganization of the polymer crystalline phases, strong embrittlement and an increase in hydrophilicity. The PBS/PLA 70/30 blend exhibited increased resistance to degradation with respect to the neat PLA and PBS films. In C&D conditions, no microparticles were observed up to 12 weeks of degradation.

## 1. Introduction

The environmental pollution generated by accumulation of synthetic plastic wastes and debris in terrestrial and aquatic ecosystems has raised numerous concerns in the last years and has recently been the subject of numerous studies [1,2,3,4,5].

The main source of plastic waste derives from packaging materials, including flexible films and rigid containers. In fact, the plastic packaging market is the largest one for plastic consumption worldwide [6,7,8,9]. In particular, plastics represent the largest, most harmful and most persistent fraction of aquatic litter: it is estimated that every day, the equivalent of more than 2000 plastic-filled garbage trucks are dumped into our oceans, rivers and lakes [7,10].

Currently, the replacement of fossil fuel-based plastics by biodegradable polymers, such as polylactic acid (PLA), polybutylene succinate (PBS), polyhydroxyalkanoates (PHA), poly (butylene adipate-co-terephthalate) (PBAT), cellulose acetate, polycaprolactone (PCL) and poly(vinyl alcohol) (PVOH) has been encouraged as a possible solution to reduce environmental issues [11,12,13]. Today, they are increasingly used as disposable packaging, mainly in the form of films [14,15,16]. However, unlike conventional fossil-derived polymers, biodegradable polymers often lack functional performance suitable for large-scale applications, such as in food packaging [17,18,19,20,21]. To improve their functional properties and expand their commercial uptake in the packaging field, biodegradable polymers are often mixed with the appropriate additives (i.e., active and smart agents, inorganic and organic nanoparticles, processing aids, metals, biomolecules from food wastes valorization, etc.) [22,23,24,25,26,27,28,29] or blended with other biodegradable polymers [30,31,32,33,34].

Proper management of post-consumer biodegradable polymers and their blends involves disposal through industrial composting sites, which break them down into low-molecular-weight compounds (water, methane, carbon dioxide) in a relatively short time under specific biotic and abiotic conditions (temperature, humidity, pH, solar radiation, bio-surfactants, presence of microorganisms and enzymes) [35,36,37]. The degradation rate of biodegradable polymers also depends on intrinsic material factors, such as number and type of functional groups, molecular weight, crystallinity degree, shape and size [38,39]. When biodegradable polymers enter the natural environment, their behavior becomes like that of non-biodegradable materials: this entails long-term decomposition through photodegradation, biodegradation and hydrolysis mechanisms, and generation of large amounts of bio-microplastics (BioMPs, ≤5 mm) which can be persistent and remain for decades, posing a real ecological pollution risk [35,40,41,42]. BioMPs can influence plant development, change the soil microbiota and affect the antioxidant system of fish and shellfish [43,44,45,46,47]. For biodegradable polymer blends, factors such as phase compatibility, interfacial interactions, as well as changes in morphology, wettability, mechanical properties, and surface charge induced by blending may result in an increased or decreased resistance to degradation and formation of BMPs [30,48,49]. As a matter of fact, the degradation behavior of a polymer blend may differ substantially from the degradation pathways of the pure components, as interactions between the different species in the blends and between the degradation products can occur during degradation. For this reason, the additive rule cannot often be applied in the case of degradation of polymer blends and, therefore, it is hard to foresee the degradative behavior of a polymer blend based on the properties of the pure components [50].

To our best knowledge, almost no work in the literature investigated the degradation of biodegradable polymer blends and the formation of BMPs from biodegradable packaging films in natural ecosystems, especially in aquatic ones. Among the studies on biodegradable films, previous literature focused on the degradation of neat films made by poly(ε-caprolactone) in a buffer–enzymatic solution [35,51,52], or on the thermal degradation of PLA-based blends [53,54]. Among biodegradable blends, only Zhao et al. addressed the photodegradation of a commercial blend based on polylactic acid/poly(butylene adipate-co-terephthalate)/thermoplastic starch (PLA/PBAT/TPS) in seawater; however, they prepared pristine microparticles of the blend by mechanical grinding, eventually subjected to UV irradiation in seawater [48].

In this scenario, the article aims at exploring the potential formation of microplastics from biodegradable packaging films based on PLA, PBS and a PBS/PLA 70/30wt% blend in different aquatic environments, and to understand the underlying mechanisms. PLA and PBS were chosen as they are among the most widely used biopolymers for flexible food packaging, thanks to their good processability and chemical–physical and functional properties, which are further enhanced by blending. The tests were carried out in four aquatic media, namely natural seawater, fresh water, sterilized seawater and sterilized fresh water, in the attempt to discriminate the contribution of water composition and microbial content on the degradation and BMPs formation. Two different conditions of temperature and light exposure were established during the tests: in the first condition, the films were kept at a temperature equal to 28 °C and exposed to a LED plant culture lamp for 14 h/day in order to simulate the ageing of plastic items immersed in water and exposed to sunlight; in the second condition, the films were kept at a temperature of 4 °C in darkness to simulate a deep-water environment. The tests were carried out up to 12 weeks. The pH changes in the aqueous environments were evaluated, while the morphology, size, wettability, crystallinity and mechanical properties of the formed microplastics were studied, analyzing the changes induced by polymer blending in the degradation behavior with respect to the neat polymers.

## 2. Materials and Methods

### 2.1. Materials, Water Collection and Analysis

PLA 4032D (semicrystalline, D-isomer content = 1.5 wt%, Mw ~155,000 g/mol, density = 1.24 g/cm^3^, Tm = 155–170 °C) was supplied by NatureWorks (Minnesota, MN, USA).

BioPBS FZ91PM (MFR (190° C, 2.16 kg) = 5 g/10 min, density = 1.26 g/cm^3^, Tm = 115 °C) was supplied by Mitsubishi Chemical Co. (Tokyo, Japan). Both the biodegradable polymers are suitable for food contact in conformity with regulations established by the U.S. Food and Drug Administration (FDA) and the European Union (EU). They also comply with EN13432 and ASTM D 6400 standards regarding compostability under controlled composting conditions. 

Tap water from the system of the University of Salerno was used as freshwater (FW). Seawater (SW) was collected from the Tyrrhenian Sea in Vietri sul Mare, province of Salerno, Italy. Their main chemical–physical parameters were measured and are reported in Table S1. pH and conductivity measurements were conducted using the GPL 21 benchtop pH meter (Crison Instruments, Barcelona, Spain) equipped with a Sension+ 5010T pH electrode (Hach Lange, Düsseldorf, Germany) and an Orion Star™ A212 benchtop conductivity meter (Thermo Scientific, Waltham, Massachusetts, USA), respectively. Alkalinity, hardness and chlorides were determined by the Lovibond^®^ MD610 benchtop photometer (Tintometer GmbH, Dortmund, Germany) equipped with reagent drop test kits. Total dissolved solids were calculated according to Rusydi [55]. Sterilized fresh water (SFW) and sterilized seawater (SSW) were obtained by autoclave sterilization at 121 °C for 20 min. All other chemicals used were of analytical grade and supplied by Sigma-Aldrich (Milan, Italy).

### 2.2. Blend and Films Preparation

Before processing, PLA and PBS pellets were vacuum dried at 70 °C for 14 h. The PBS and PLA4032D blend, with mass ratio 70/30 by weight, was prepared in a co-rotating twin screw extruder (Collin ZK25, with screw diameter equal to 25 mm and L/D = 42), with a screw speed equal to 150 rpm and a temperature profile from 150 °C to 190 °C from the hopper to the die. Monolayer films consisting of neat PLA, neat PBS and PBS/PLA blend were made by a lab-scale film blowing plant equipped by a single screw extruder (GIMAC, D = 12 mm, L/D = 24), setting the same temperature profile as described above and the blow-up ratio at 2.5. The collection speed was fixed at 3 m/min, yielding samples with an average thickness of 50 ± 2 µm.

### 2.3. Degradation Experiments

**Figure 1:**
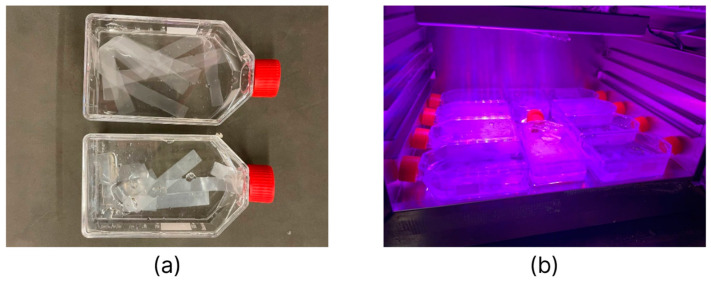
Experimental conditions: (**a**) samples in the flask; (**b**) flasks in the climate chamber.

Degradation tests were carried out on 12.7 mm × 150 mm film cut strips that were placed in Greiner T-75 cell culture flasks with a canted neck, a ventilated cap, a cross-section surface area of 75 cm^2^ and a capacity of 250 mL, as shown in Figure 1a. Each flask was filled with 12 film strips and 100 mL of aquatic medium, either fresh water (FW), fresh sterilized water (SFW), seawater (SW), or sterilized seawater (SSW). 

Two different conditions of temperature and light exposure were established during the tests. In the first one (named Warm&Light—W&L), the flasks were kept in a conditioned chamber at 50% RH and 28 ± 1 °C under LED plant culture lamps (MOSOTON MT-102 25 W LED panel, Mosoton, Guangzhou, China) with 14 h of light and 10 h of darkness each day (Figure 1b) in order to simulate the ageing of the plastic items immersed in water and exposed to sunlight. The flasks were shaken twice a week to improve oxygen supply in the medium and exchanged positions to ensure homogeneity of light exposure for all samples. In the second condition (named Cold&Dark—C&D), the flasks were kept in a refrigerator for 12 weeks at 4 ± 1 °C, and culture flasks were wrapped in aluminum foil to avoid light detection, in order to simulate a deep-water environment. 

The tests were conducted for up to 12 weeks, and the samples were analyzed at time 0 and after 5, 7 and 12 weeks of storage for the W&L condition, and after 12 weeks for the C&D condition. At each time interval, three replicate flasks for each type of biodegradable film and degradation condition were withdrawn. Then, the exposed film strips were removed, washed in an ultrasonic bath for 5 min with Tergazyme detergent at 3% in warm water to remove the biofilm, rinsed with distilled water, patted dry with a lint-free cloth and equilibrated in a climate chamber at controlled conditions (23 °C and 50% RH) for 24 h before the analyses. The remaining culture flasks with water were stored in a refrigerator at a temperature of 4 ± 1 °C before further tests.

### 2.4. Characterizations

pH changes occurring in the different aqueous media during degradation were measured using a GPL 21 benchtop pH meter (Crison Instruments, Barcelona, Spain) equipped with a Sension+ 5010T pH electrode (Hach Lange, Düsseldorf, Germany), with pH accuracy equal to 0.02.

The shape and average size of the formed microplastics were analyzed using an optical microscope (Zeiss Axioskop 40, Carl Zeiss, Oberkochen, Germany) equipped with a Axiocam 208 color camera. The analyses were performed on droplets (V = 0.5 mL) of the different aqueous media, taken from the flasks, pipetted on microscope slides and dried at room temperature for 1 day in a vacuum oven. 

Thermal analyses of the samples were carried out by a differential scanning calorimeter DSC PT 1000 (Linseis, Selb, Germany). The heating/cooling thermal cycle was set at a rate of 10 °C/min in the range from 25 to 200 °C under a nitrogen gas flow (20 mL/min). The crystallinity degree of each component *i* (i.e., PBS and PLA) of the films was calculated according to the following equation:

(1) $$X_{c_{i}}=({∆H}_{m_{i}}-{∆H}_{{cc}_{i}})/(\phi_{i}\times {∆H}_{m_{i}}^{0})$$

where ${∆H}_{m_{i}}$ and ${∆H}_{{cc}_{i}}$ are the melting and the cold crystallization enthalpies of component *i* in the film, respectively; ${∆H}_{m_{i}}^{0}$ is the melting enthalpy of component *i* 100% crystalline (equal to 110.3 J/g for PBS and 93.7 J/g for PLA); and $\phi_{i}$ is the weight fraction of component *i* in the film. 

The film strips were submitted to tensile testing according to the ASTM D 882-91 procedure, using a Sans CMT6000 dynamometer (Shenzhen, China) equipped with a 100 N load cell. Tensile tests were carried out at 23 °C and 50% RH, setting the crossbar speed at 3 mm/min to evaluate the elastic modulus, and at 300 mm/min to evaluate strength and elongation at break. The results were expressed as the average of at least seven measurements taken for each type of film and water medium.

Static contact angle measurements using the sessile drop method were recorded and analyzed at room temperature using an FTA 1000 analyzer (First Ten Angstroms, Inc., Portsmouth, VA, USA) according to ASTM D 7490. A 2 ± 0.5 µL drop of distilled water was applied to the sample surface using a syringe. The drop’s image was captured by a video camera immediately after deposition, and the contact angle was calculated from the drop’s shape using proprietary image analysis software. The reported contact angles are the average of at least ten replicate measurements. 

## 3. Results and Discussion

### 3.1. pH Changes of the Aquatic Media

**Figure 2:**
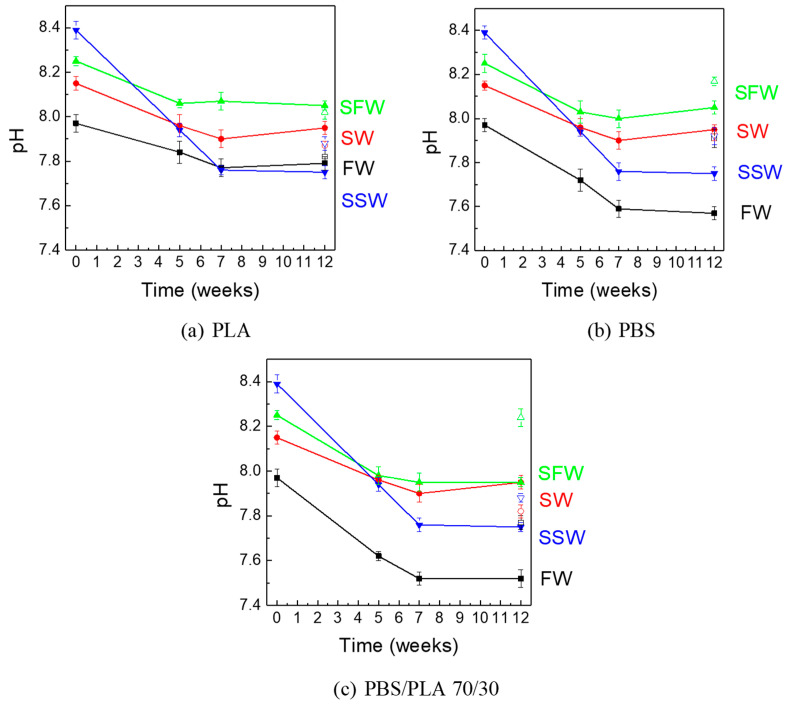
Measured pH values in the aqueous media (FW, SW, SFW and SSW) in the presence of films made of (**a**) PLA, (**b**) PBS and (**c**) PBS/PLA 70/30 (full symbols: Warm and Light conditions; empty symbols: Cold and Dark conditions).

The pH changes of the aquatic media with time, both in W&L and C&D conditions, were monitored as an indirect indication of the film degradation progress in the different aqueous environments. It is known, in fact, that the hydrolysis of the ester bonds of aliphatic polyesters decreases the molecular weight because of molecular chain fracture and leads to the formation of short-chain monomers and oligomers with a weakly acidic character [56]. The results of the measurements are reported in Figure 2 and Table 1.

At the beginning, all aqueous media had weak alkaline pH values. The chemical potential of water plays a significant role in the hydrolysis of polyesters: in fact, hydrolysis in alkaline conditions is faster than in acidic ones [57]. FW initially had a pH of 7.97, while SW showed a pH equal to 8.15; these values are consistent with those reported in the literature [58]. Sterilized freshwater (SFW) and seawater (SW) had an initial pH equal to 8.25 and 8.39, respectively. In all cases, the pH values remained essentially constant with time in control samples.

In the W&L condition, a decrease in pH with respect to the initial value was observed during exposure to all the aqueous media with all the film samples. After 12 weeks, for FW, SW and SFW, the average pH decrease ranged within 0.18–0.20 units for the PLA film, 0.20–0.40 units for the PBS film and 0.28–0.45 units for the PBS/PLA 70/30 film. For all the tested films, the most consistent pH decrease was observed in SSW: at the end of the test, pH decreased by 0.65 units for the PLA film and by 0.70 units for PBS and PBS/PLA 70/30 samples. The observed acidification of the aqueous environments can be reasonably attributed to the release of monomer and oligomer byproducts, such as lactic acid as well as 4-hydroxybutyl succinate and succinic acid formed during the hydrolytic degradation of the PLA and PBS polymers, respectively [59]. The largest decrease in pH obtained for SSW could be explained by the fact that the sterilization of seawater destroys microorganisms capable of metabolizing PLA and PBS, as reported elsewhere [60,61,62,63,64]. Similar outcomes were gained for the tests in C&D conditions, although to a slightly smaller extent than in W&L conditions, as expected. In fact, the degradation kinetic of biopolymers is made faster by increasing temperature and the presence of light radiation, as largely reported in literature [59,65].

Overall, the obtained results are consistent with those reported by Romera-Castillo et al. [66], who observed an average pH decrease of 0.49 units due to biodegradable and non-biodegradable plastic leaching in seawater and emphasized the risk of exacerbating the harmful effects on marine organisms of acidification resulting from anthropogenic CO_2_ emissions [67].

### 3.2. Optical Microscopy Analysis of the Formed Microplastics

Optical microscopy analysis was used to obtain information on the size and shape of the microparticles formed from PLA, PBS and PBS/PLA 70/30 films, after immersion in the four different aquatic environments in W&L conditions for 5, 7 and 12 weeks. Since the microscopy observations revealed no fragment formation in any case even after 12 weeks of exposure in C&D conditions and no appreciable differences between the same aquatic media in W&L conditions, sterilized and non-sterilized, only the images taken on the non-sterile fresh and seawater in W&L conditions are reported below.

**Figure 3:**
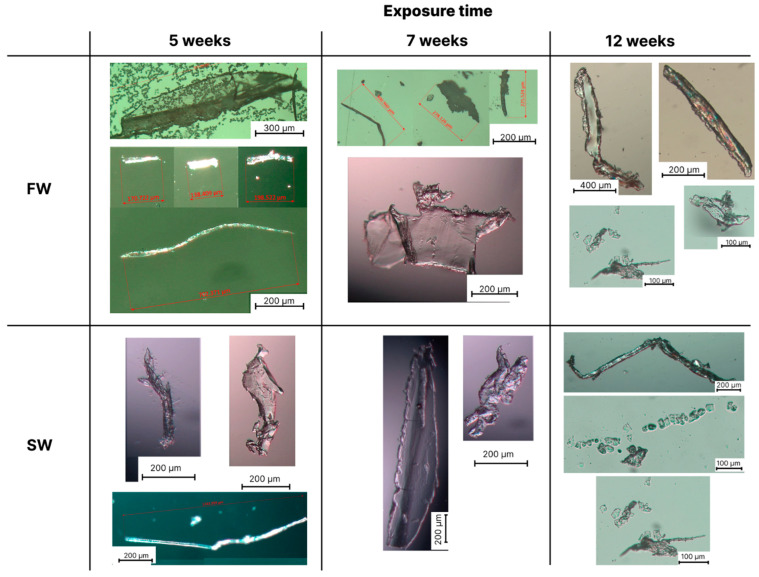
Optical microscopy images of the formed microplastics formed after 5, 7 and 12 weeks of degradation of PLA films in fresh water (FW) and seawater (SW). Black bars represent the reference size. Red bars represent the average measured particle length.

**Figure 4:**
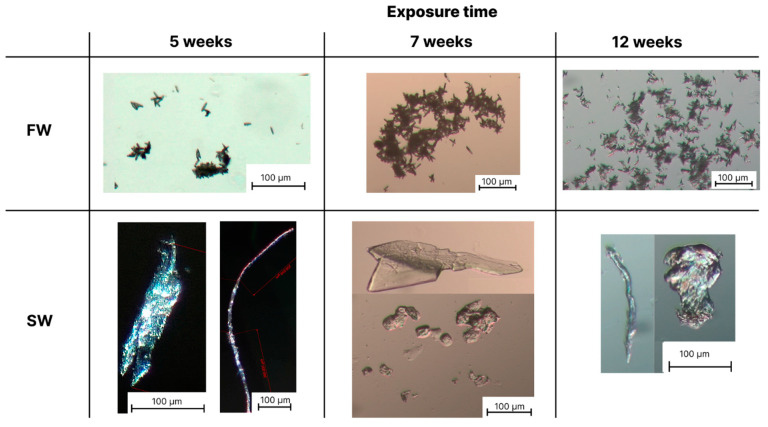
Optical microscopy images of microplastics formed after 5, 7 and 12 weeks of degradation of PBS films in fresh water (FW) and seawater (SW). Black bars represent the reference size. Red bars represent the average measured particle length.

**Figure 5:**
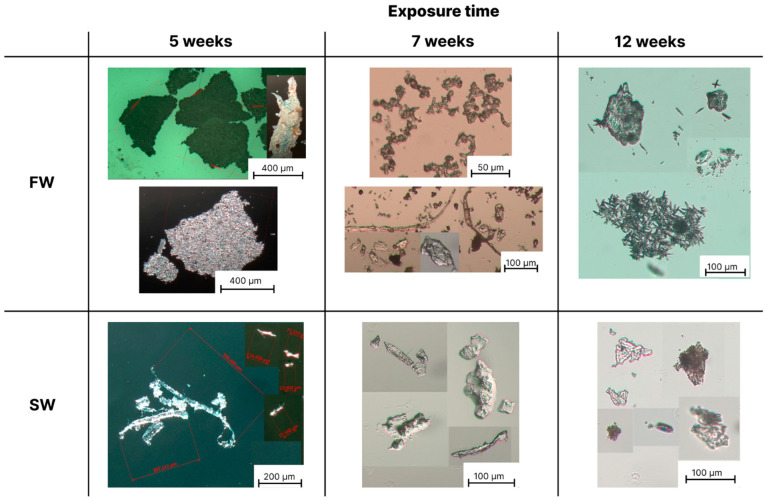
Optical microscopy images of microplastics formed after 5, 7 and 12 weeks of degradation of PBS/PLA 70/30 films in fresh water (FW) and seawater (SW). Black bars represent the reference size. Red bars represent the average measured particle length.

Figure 3, Figure 4 and Figure 5 show the photos of microplastics from PLA, PBS and PBS/PLA 70/30 films, respectively, after different immersion times in FW and SW.

For the PLA film (Figure 3), a large number of fragments was observed after 5 weeks in all the aqueous environments investigated, with similar shape and average size. In particular, the presence of either fibers, which constituted the most abundant fraction, with a length up to 700 µm, or platelets, with an average length up to 1.5 mm, was evident. After 7 weeks, BMPs of similar shape and size were observed compared to those obtained after 5 weeks. After 12 weeks, some longer fiber-like structures, up to 1.5 mm, were also found, while the average size of the platelets decreased to 300 µm. It was not possible to discriminate significant differences in the production of BMPs in the different aqueous media due to the presence of microorganisms in the unsterilized media or to differences in ionic strength. This result suggests that the PLA films predominantly underwent an abiotic degradation in all investigated aqueous media, with essentially comparable kinetics; as also reported by [59,68], in fact, hydrolytic degradation is predominant for aliphatic polyesters, such as PLA and PBS, following a bulk erosion mechanism for thicknesses between 0.5 and 2 mm.

As regards the PBS film (Figure 4), after 5 weeks, the production of few acicular microplastics in FW and SFW was observed, having an average size of 20 μm. By increasing the exposure time to 7 and 12 weeks, the number of these particles steadily increased. Interestingly, a large number of BMPs entangled together and formed large fragments, with a length of up to 500 μm. This peculiar morphology was only found for FW and SFW. For SW and SSW, mostly fibers with 1 mm maximum length, and platelets with 500 μm maximum size were noted after 5 weeks; after 7 and 12 weeks, the maximum size for both fibers and platelets decreased to 300 μm. Additionally, in this case, no difference was appreciable due to the presence of microorganisms.

It is worth to note that, from the conducted observations, most of the plastic microparticles obtained for PBS and PLA films fell into the fiber category. BMPs with fiber-like structure and size up to 2 mm were also observed by Wei et al. for PBAT films after 10 weeks of exposure in artificial seawater and Milli-Q water [35]. In accordance with what reported by Hebner et al., the preferential formation of microplastic fibers rather than platelets can be attributed to the processing of the polymer films, which imparts orientation to the macromolecules [69]. The penetration of water in thin films further exacerbates the creation of oriented cracks that lead to the specimen’s breakage along the direction of alignment of the polymer macromolecules, resulting in fiber-like particles rather than platelets.

As regards the PBS/PLA 70/30 film (Figure 5), the formation of microplastics was much more heterogeneous than in the pure films. In FW and SFW, large heterogeneous particles with size up to 800 µm were observed after 5 weeks of exposure, as well as platelets up to 1 mm, consisting of aggregates of smaller particles like in the case of pure PBS (Figure 5). After 7 weeks of exposure, similarly to neat PBS film, the micrographs also revealed the presence of 20 μm-long acicular particles, alone or aggregated in bigger clusters. After 12 weeks, fibers up to 500 µm and acicular clusters up to 100 µm were observed. In SW and SSW, after 5 weeks of exposure, fiber-shaped particles up to 600 µm in length were found, as well as small particles of inhomogeneous shape up to 100 µm (Figure 5). After 7 and 12 weeks, BMPs mainly consisted of platelets up to 500 µm, fibers up to 200 µm and inhomogeneous particles up to 100 microns (Figure 5).

The resulting outcomes, compared with those achieved for pure PLA and PBS films, suggest a slower degradation rate for the PBS/PLA 70/30 blend, at least initially, with the formation of larger and more heterogeneous particles. As exposure time increased, the formation of microplastics having the characteristic shape and size of both the PBS and PLA phases was observed. The degradation rate of the blend was largely influenced by the composition of the blend, phase compatibility, degree of crystallinity, wettability and mechanical properties achieved. These characteristics will be discussed further in the article. For PBS/PLA mixtures, studies in the literature reveal very different degradation behaviors depending on these parameters. For example, Luzi et al. in their study observed a decreased degradation rate for the PLA/PBS 80/20 blend compared to that of neat polymers because of the higher degree of crystallinity induced by PBS [70]. On the other hand, Wang et al. found that immiscibility between PLA and PBS induces gaps in the mixture, providing channels for water penetration and enhancing hydrolytic degradation [71].

### 3.3. Melting Behavior, Crystallinity and Glass Transition Temperature

DSC measurements were carried out to investigate the initial film morphology and crystallinity, which deeply affect the degradation rate, and to study the changes in the thermal behavior of the polymer phases during the degradation processes.

**Figure 6:**
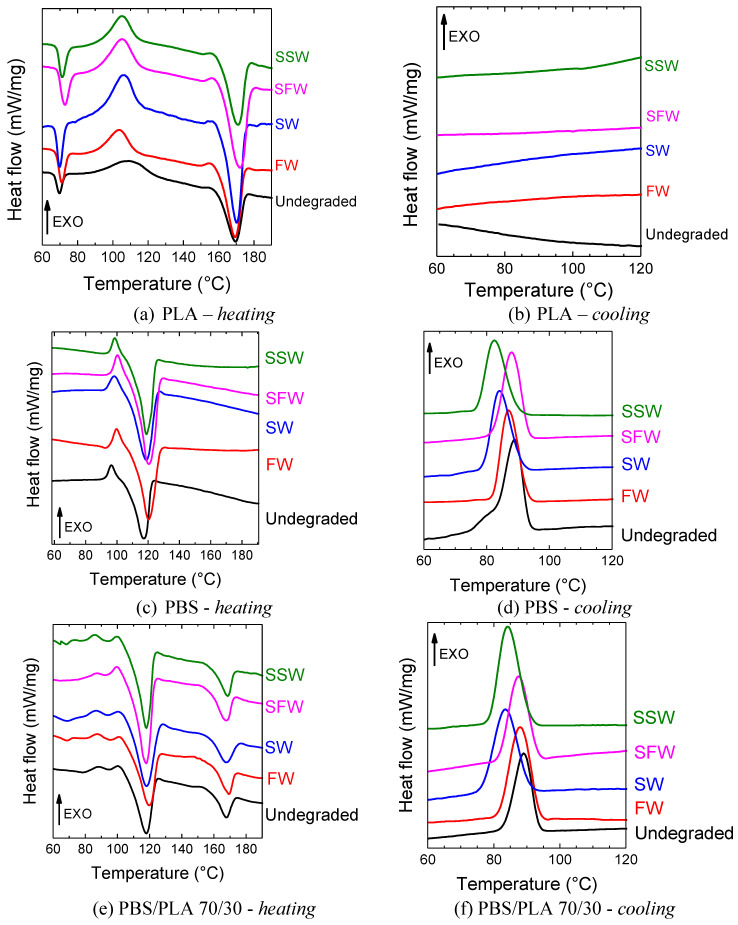
DSC thermograms of the PLA, PBS and PBS/PLA 70/30 films as produced and after 12 weeks of degradation in aqueous media in the Warm and Light condition.

Figure 6 displays the heating and cooling thermograms of the PLA, PBS and PBS/PLA 70/30 blend films, undegraded and after 12 weeks of degradation in the W&L condition in the four aqueous media; the corresponding thermal parameters are shown in Table 2.

Regarding the PLA film, before degradation, it exhibited a glass transition temperature at 67 °C, a broad cold crystallization peak in the range 100–120 °C, a second small exothermic event just before melting that, according to the literature, can be related to the reorganization of the PLA α’-mesophase into more stable α-crystals [72], and a melting peak at 169 °C. The crystallinity degree was equal to 12.8%. After 12 weeks of exposure in aqueous media, the *Tg* slightly raised from 68 up to a maximum of 71 °C. This increase of *Tg* values has been previously reported by other authors [73,74] and can be attributable to the stabilized packing of the chains in the amorphous region by annealing in the presence of water molecules. This raise in *Tg* leads to further embrittlement of the PLA sample, which is one of the most detrimental effects of degradation and accelerates film fragmentation. The specific interactions between water and PLA polymer chains do not only promote order and stability in the amorphous region but also in the crystalline region, as highlighted by the slight increase in the melting temperature and the crystallinity of the PLA films, up to maximum *Tm* = 173 °C and *Xc* = 19.5% after 12 weeks in SFW. These changes can be related to the formation of thicker, more perfect crystals with a higher melting temperature [75]. No peaks were found in the cooling scans in all cases, as expected, due to the low crystallization rate of PLA [76].

Concerning the PBS film, before degradation, the DSC trace showed a small cold crystallization peak at 96 °C and a melting transition at 118 °C, giving a crystallinity degree equal to 56.4%. After degradation experiments, the cold crystallization temperatures tended to increase. In fact, the lowering of the molecular weight caused by molecular chain fracture affected the cold crystallization kinetic, which became slower, as found also by others [77,78]. However, the corresponding *ΔH_cc_* values remained almost the same. No relevant changes in the crystallinity degree of the films after degradation were detected, too. In the cooling scans, the crystallization peak became broader and shifted towards lower temperatures for all the films biodegraded in the aqueous media. A similar trend has been reported in the literature for dynamic crystallization from melt of a different polyester with decreasing molecular weights and is coherent with the hydrolytic degradation of PBS [79]. The changes are more relevant in both the seawater media, suggesting that the PBS hydrolysis rate can be enhanced in high-ionic-strength media, such as marine solutions [35,80].

As for the PBS/PLA 70/30 blend, before degradation, the thermogram showed a first small cold crystallization peak at 88 °C, related to PBS, another small exotherm transition at ca. 100 °C attributable to PLA just before the PBS melting peak at 118 °C, and a second melting peak at 167 °C due to PLA. The two well-distinct melting peaks confirmed the complete phase separation of the two polymers. Due to the overlapping of the cold crystallization peak of PLA with the melting peak of PBS, it was difficult to determine the cold crystallization enthalpy and thus the crystallinity of PLA in the blend. However, following the same approach described in [32], it was possible to calculate this value with a slight bias: the crystallinity degrees of PBS and PLA phases in the blends, equal to 64.4% and 52.3%, respectively, revealed a substantial increase in the crystallinity degree of the PLA phase with respect to the neat PLA film, ascribable to the higher mobility of the PLA chains in the presence of PBS, with an increased ability to crystallize. 

This suggests an increase in the resistance to hydrolytic degradation of the PBS/PLA 70/30 blend compared to that of pure PLA and PBS films, as already observed by Luzi et al., and is corroborated by optical microscope observations, which revealed the presence of larger and more heterogeneously shaped fragments compared to the microplastics produced by pure polymer films [70]. With regard to the cooling thermograms, similarly to what was observed for the pure PBS film, a shift of the crystallization temperatures ascribable to the PBS phase towards lower values was observed after degradation exposure in SW and SSW (Tc equal to 89, 84 and 82 °C for the initial film and after exposure in SW and SSW, respectively), ascribable also in this case to the shortening of the PBS chains due to hydrolytic degradation.

### 3.4. Mechanical Properties

**Figure 7:**
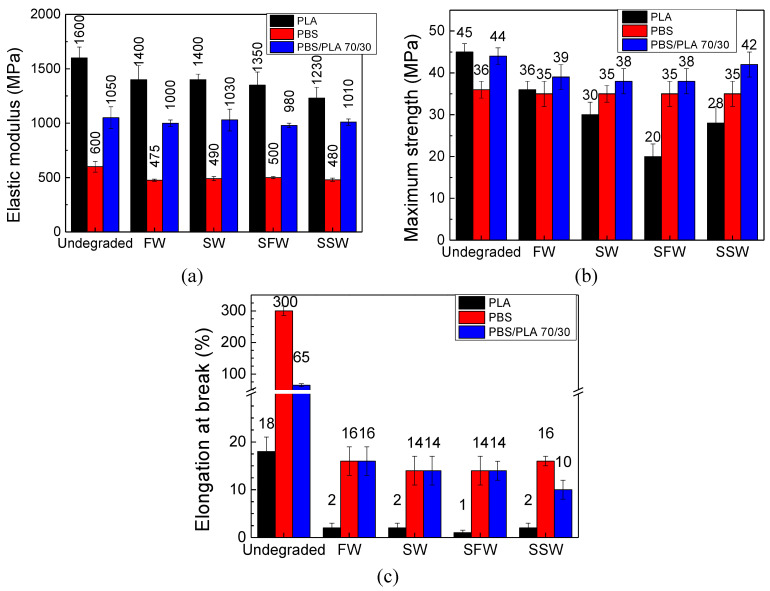
Mechanical properties of films after 7 weeks of degradation in Warm and Light (W&L) conditions: (**a**) elastic modulus E; (**b**) maximum strength σ_m_; (**c**) elongation at break ε_b_.

Tensile tests were carried out to reveal changes in the mechanical properties of the PLA, PBS and PBS/PLA 70/30 films during exposure in aquatic environments. This represents an effective methodology for investigating the degradation rate of the biodegradable films [60]. The results, expressed as elastic modulus E, maximum strength σ_m_ and elongation at break ε_b_, are displayed in Figure 7 for the W&L condition in the four aqueous media. It is worth pointing out that, since the PLA films experienced fast degradation kinetics, as also underlined by optical microscopy and DSC analyses, their tensile properties could be measured only up to 7 weeks of exposure; after 12 weeks, their advanced state of degradation did not allow to subject them to tensile testing. For this reason, in order to make a comparison between the mechanical properties of PLA, PBS and PBS/PLA 70/30 films, the results presented were collected after 7 weeks of exposure for all the samples. The results of tensile tests on PBS and PBS/PLA 70/30 film strips after 12 weeks of degradation in W&L condition are available in the Supplementary Material.

The mechanical properties obtained at the initial time confirmed the inherent stiffness and brittleness of the PLA film (E = 1600 ± 100 MPa, σ_m_ = 45 ± 3 MPa, ε_b_ = 18 ± 3%), while PBS showed very ductile behavior (E = 600 ± 50 MPa, σ_m_ = 36 ± 2 MPa, ε_b_ = 300 ± 20%), as also reported by other authors [81,82]. The PBS/PLA 70/30 film exhibited a good balance between stiffness and ductility (E = 1050 ± 70 MPa, σ_m_ = 44 ± 3 MPa, ε_b_ = 65 ± 5%). 

A decrease in the tensile properties was observed for all the films after exposure in the aqueous media, especially regarding elongation at break, which is the most sensitive index for monitoring the cleavage of polymer chains [83]. However, some relevant differences could be noticed based on the composition of the films, rather than on the presence of microorganisms or ionic strength.

After 7 weeks, the PLA film underwent the most severe embrittlement and decrease in stiffness: with respect to initial time, in the four aqueous media, the decrease in elastic modulus, elongation at break and maximum tensile strength ranged from 13% to 23%, from 88% to 95% and from 20% to 55%, respectively. The results obtained for the PBS film also demonstrated a transition from ductile to brittle behavior upon hydrolytic degradation; however, to a lesser extent than in the PLA sample: the decreases in E, ε_b_ and σ_m_ ranged from 16% to 21%, from 71% to 94% and from 2% to 3%, respectively. As underlined by DSC analyses, the increased resistance to hydrolysis can be attributable to the higher crystallinity degree of PBS with respect to PLA. The PBS/PLA 70/30 film exhibited the lowest embrittlement and stiffness reduction: the decrease in E, ε_b_ and σ_m_ ranged from 3% to 6%, from 77% to 84% and from 2% to 13%, respectively. These results further confirm the outcomes gained by DSC and optical microscopy analyses, which underlined the increased resistance to hydrolytic degradation of the PBS/PLA blend with respect to that of their parent polymers. Different outcomes were obtained by Zhou et al., who observed that PBS/PLA blends lost their tensile properties earlier than their parent polymers with the proceeding of hydrolysis [84]. Other authors analyzed the effect of hydrolytic degradation on the mechanical properties of fossil-based polyesters such as polyethylene terephthalate and highlighted that during hydrolysis, an embrittlement of the polymer is observed that leads to a large decrease in both strain and stress at break, whereas Young’s modulus remains almost constant [85].

**Figure 8:**
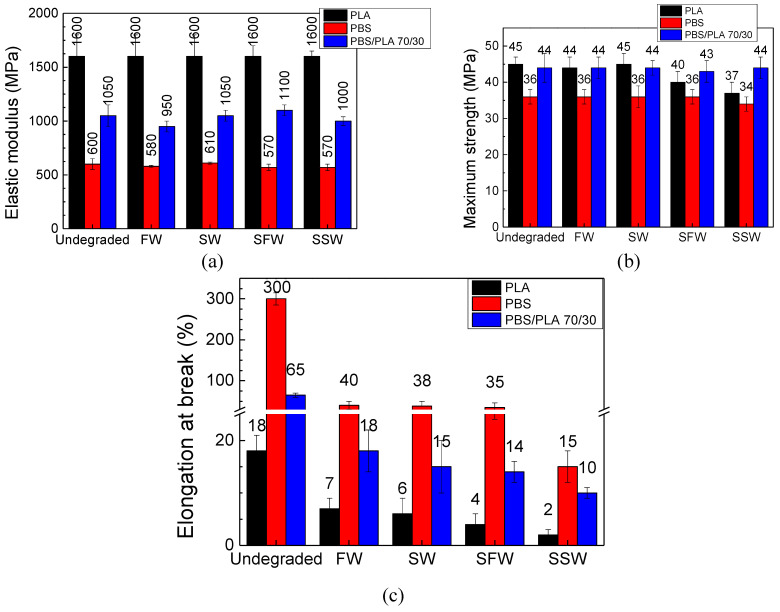
Mechanical properties of films after the 12 weeks of degradation in Cold and Dark (C&D) conditions: (**a**) elastic modulus E; (**b**) maximum strength σ_m_; (**c**) elongation at break ε_b_.

The mechanical properties of the specimens after 12 weeks under C&D conditions are shown in Figure 8. It can be noted that under these conditions, the specimens retained their strength and modulus of elasticity regardless of the water environment; however, an increase in the brittleness of the specimens was observed, but to a lower extent than that measured in W&L conditions. These results are in accordance with the outcomes gained from the optical microscopy analysis and could be attributable to slower water diffusion into the samples under these conditions: this points out that, in cold deep water, biodegradable plastics can be highly persistent and could behave like non-degradable polymers.

### 3.5. Surface Wettability

Water contact angle measurements were carried out to evaluate changes in surface wettability of the biodegradable films during degradation in the aqueous media in Warm and Light (W&L) conditions. Table 3 shows the values of the water contact angle for PLA, PBS and PBS/PLA 70/30 films, both undegraded and after 12 weeks of degradation in FW, SW, SFW and SSW.

For all the materials and the aqueous media, the wetting angle decreased after 12 weeks of exposure, implying an increase in hydrophilicity. The PLA film exhibited the maximum decrease in contact angle equal to 36% in FW, while for PBS and PBS/PLA 70/30, maximum decreases equal to 31% in SSW and 23% in FW, respectively, were registered. The increase in hydrophilicity could be attributed to both the increase of surface roughness due to swelling and erosion phenomena and to the cleavage of the ester bonds occurring during hydrolytic degradation, with the accumulation of polar low-molecular-weight substances on the surface of the films. In particular, PLA degradation entails the formation of as carboxylic acids (e.g., lactic acid) and hydroxyl groups [86], while the main products of PBS hydrolytic degradation are 4-hydroxybutyl succinate, succinic acid and butane-1,4-diol [87]. Compared to that of the PLA film, the smaller decrease in water contact angle of the PBS and PBS/PLA 70/30 films could represent an indirect measurement of the increased resistance to hydrolytic degradation of these samples, which involved the generation of lower amounts of water-soluble oligomers and monomers.

## 4. Conclusions

In this work, the risk associated with the production of BMPs from biodegradable packaging was investigated by monitoring the deterioration behaviors of PLA, PBS and PBS/PLA 70/30 blend films for 12 weeks in different aquatic media and two temperature/light conditions.

Among the different biodegradable polymers, PLA underwent the most rapid degradation, particularly in W&L tests. In this condition, the PLA film, due to water swelling that increased the chain mobility, underwent to both solvent-induced crystallization, shown by DSC results, and hydrolytic degradation, evidenced by the dramatic film embrittlement (with a ε_b_ decrease in the range ca. 88–94%), which is the most sensitive index for monitoring the cleavage of polymer chains. Therefore, a high number of BMPs was generated already after 5 weeks, mostly consisting of fibers up to 700 µm long. After 12 weeks of exposure, the PLA film completely lost its integrity. On the other hand, the PBS film, thanks to its intrinsic high crystallinity degree, had a much slower degradation rate, exhibiting only slight and insignificant modifications of its thermal, tensile and surface properties, and produced only few acicular BMPs after 5 weeks, and more fibrous BMPs having 300 to 500 µm average size for longer exposure times. Lastly, the PBS/PLA 70/30 blend exhibited the highest resistance to deterioration, attributable to the higher crystallinity degree of the PLA phase in the blend (52.3%) with respect to that of the neat PLA film (12.8%). Compared to its parent polymers, the blend showed the lowest embrittlement (maximum εb decrease equal to 84%) and the lowest increase in hydrophilicity, and its hydrolytic degradation led to the formation of large and quite heterogeneous BMPs, consisting of acicular clusters, fibers and inhomogeneous platelets, whose sizes ranged from 100 to 800 µm. These findings pointed out that polymer blends may pose a greater risk to aquatic environments than neat polymers, and caution should be adopted when encouraging biodegradable polymer mixtures.

Only minor changes were detectable in degradation behavior of the films due to the different ionic strength of aqueous media and to the presence of microorganisms. However, all the aqueous environments incurred acidification during the degradation experiments, and a larger decrease in pH was observed in sterilized media (i.e., SFW and SSW) than in unsterilized ones (i.e., FW and SW), suggesting that microorganisms could absorb acidic degradation byproducts generated during polyester hydrolysis.

Finally, the results pointed out that temperature and light exposure severely affected the degradation rate of the bioplastics, which can be highly persistent in cold deep water, behaving like non-degradable polymers.

## Figures and Tables

**Table 1:** Differences between initial pH and pH after 12 weeks of the four aqueous media: fresh water (FW), seawater (SW), sterilized fresh water (SFW) and sterilized seawater (SSW), without film (control) and with PLA, PBS and PBS/PLA 70/30 films, stored under Warm and Light (W&L) and Cold and Dark (C&D) conditions.

Samples	Conditions	ΔpH (pH_week 0_–pH_week 12_)			
		FW	SFW	SW	SSW
Control	W&L	0.03	0.01	0.04	0.02
	C&D	0.02	0.01	0.03	0.02
PLA	W&L	0.18	0.20	0.20	0.65
	C&D	0.15	0.23	0.28	0.52
PBS	W&L	0.40	0.20	0.30	0.70
	C&D	0.05	0.08	0.22	0.48
PBS/PLA 70/30	W&L	0.45	0.30	0.28	0.70
	C&D	0.20	0.15	0.33	0.51

**Table 2:** Thermal parameters of the PLA, PBS and PBS/PLA 70/30 films undegraded and after 12 weeks of degradation, calculated from the first heating and the cooling scans.

Film Sample	Degradation Medium	Heating											Cooling	
		*T_g_*, [°C]	*T_cc PBS_*, [°C]	Δ*H_cc PBS_*, [mWs/mg]	*T_cc PLA_*, [°C]	Δ*H_cc PLA_*, [mWs/mg]	*T_m PBS_*, [°C]	Δ*H_m PBS_*, [mWs/mg]	*X_c_*_PBS_, [%]	*T_m PLA_*, [°C]	Δ*H_m PLA_*, [mWs/mg]	*X_c_*_PLA_, [%]	*T_cr_*, [°C]	Δ*H_cr_*, [mWs/mg]
PLA	Undegraded	68			109	25.2				169	37.2	12.8		
	FW	69			104	20.4				169	37.9	18.7		
	SW	69			106	19.6				170	36.4	17.9		
	SFW	71			105	18.6				173	36.9	19.5		
	SSW	70			106	21.7				172	37.8	17.2		
PBS	Undegraded		97	7.6			118	69.8	56.4				89	63.8
	FW		100	7.4			120	66.4	56.6				88	55.1
	SW		99	7			119	63.5	55.8				84	57.9
	SFW		100	6.9			119	70.6	60.4				88	57.5
	SSW		99	5.7			119	65.4	54.1				82	51.4
PBS/PLA 70/30	Undegraded		88	4.1	100		118	49.7	59.1	167	14.7	52.3	89	38.2
	FW		88	5.1	101		120	51.6	60.2	169	15.3	54.5	88	40.1
	SW		87	5.6	100		118	48.4	55.4	168	12.8	45.6	84	37.1
	SFW		87	3.4	100		118	51.5	62.3	168	14.4	51.2	88	39.5
	SSW		87	4.5	100		118	51.4	60.7	168	14.3	51.0	84	37.8

**Table 3:** Water contact angle values of PLA, PBS and PBS/PLA 70/30 films, both undegraded and after 12 weeks of degradation in different aqueous media in Warm and Light (W&L) conditions.

Sample	Water Contact Angle [°]				
	Undegraded	FW	SW	SFW	SSW
PLA	63.7 ± 2.5	41.0 ± 6.1	45.8 ± 6.0	53.5 ± 6.4	56.7 ± 4.0
PBS	72.5 ± 2.1	60.6 ± 2.7	53.9 ± 2.9	60.1 ± 4.8	50.2 ± 2.7
PBS/PLA 70/30	65.5 ± 3.1	50.4 ± 5.9	54.0 ± 3.9	57.5 ± 4.2	57.4 ± 5.1

## Data Availability

The data presented in this study are available on request from the corresponding author.

## Supplementary Materials

The following supporting information can be downloaded at: https://www.mdpi.com/article/10.3390/polym16162268/s1, Table S1: Chemical-physical parameters of the freshwater and seawater; Figure S1: Mechanical properties of PBS and PBS/PLA 70/30 films after the 12 weeks of degradation in Cold and Dark (C&D) conditions: a—strength at break, b—maximum strength, c—elongation at break.
