# Supplementary material for: Generation of Microplastics from Biodegradable Packaging Films Based on PLA, PBS and Their Blend in Freshwater and Seawater
Source: Polymers (Basel). 2024 Aug 10;16(16):2268. doi: 10.3390/polym16162268 (PMC11360359; doi:10.3390/polym16162268)
Supplement: Supplementary file 1 [file polymers-16-02268-s001.zip › polymers-3138588-supplementary.pdf]

Supplementary Materials

Table S1. Chemical-physical parameters of the freshwater and seawater.

Property	Freshwater	Seawater
pH	7.97	8.15
Alkalinity	193 mg/L	174 mg/L
Hardness	18 °F	> 50 °F
Chlorides	10 mg/L	24000 mg/L
Conductivity (EC)	386 μS/cm	53000 μS/cm
Total dissolved solids (TDS)	212.3 mg/L	37100 mg/L

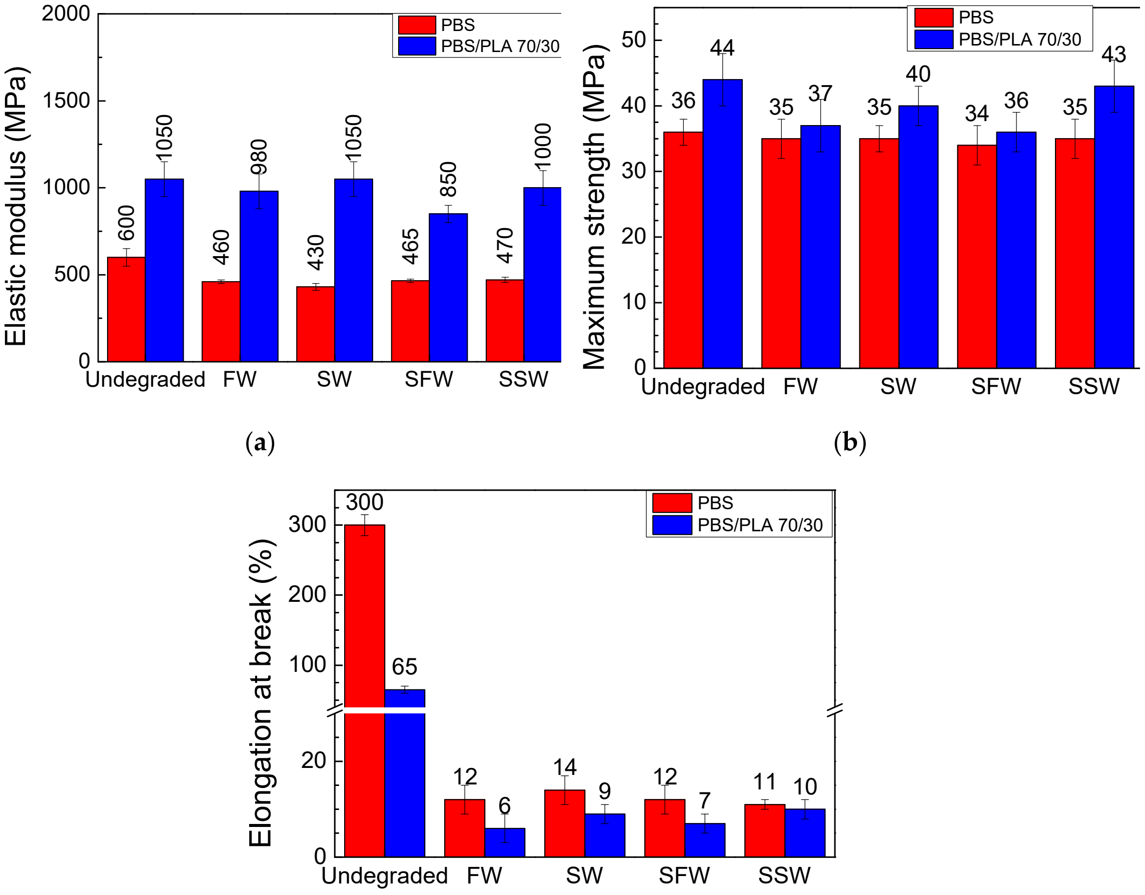

(a) (b)

(c)

Figure S1. Mechanical properties of PBS and PBS/PLA 70/30 films after the 12 weeks degradation in Cold and Dark (C&D) conditions: a—strength at break, b—maximum strength, c—elongation at break.
